# Adjuvant Chemotherapy and Survival in MSI-High Stages II and III Colon Cancer: Impact of Histopathologic Risk Stratification

**DOI:** 10.1245/s10434-025-18285-7

**Published:** 2025-10-07

**Authors:** Metincan Erkaya, Cigdem Benlice, Salih Karahan, Mustafa Oruc, Volkan Ozben, Bilgi Baca, Emre Gorgun

**Affiliations:** 1 Department of Colorectal Surgery, Digestive Disease and Surgery Institute, Cleveland Clinic, Cleveland, OH USA; 2 Faculty of Medicine, Acibadem Mehmet Ali Aydınlar University, Istanbul, Turkey

## Abstract

**Background:**

Microsatellite instability-high (MSI-H) status in colorectal cancer is associated with a favorable prognosis, but its predictive value for adjuvant chemotherapy (ACT) benefit for patients with stage II disease remains controversial, particularly when histopathologic risk factors are taken into account. This study aimed to evaluate the impact of ACT on overall survival (OS) for patients with stage II MSI-H colon cancer stratified by pathologic risk and stage III MSI-H colon cancer.

**Methods:**

A retrospective analysis of the National Cancer Database (2013–2019) was performed, identifying 8025 patients with stage II or III MSI-H colon adenocarcinoma who underwent surgical resection. The stage II patients were stratified into low- and high-risk cohorts based on adverse pathologic features including pT4 stage, lymphovascular invasion, perineural invasion, positive margins, or and fewer than 12 lymph nodes examined. Inverse probability of treatment-weighting was applied to balance the covariates, and OS was estimated using weighted Kaplan–Meier and Cox proportional hazards models.

**Results:**

Of the 8025 patients included in the study, 4981 had stage II disease and 3044 had stage III disease. Among the 3501 low-risk stage II patients, ACT was administered to only 5.9% and was not associated with an OS benefit (hazard ratio [HR], 0.92; 95% confidence interval [CI] 0.58–1.47; *p* = 0.73). In contrast, among the 1480 high-risk stage II patients, 19.1% received ACT, which was associated with a 62% reduction in mortality risk (HR, 0.38; 95% CI 0.25–0.57; *p* < 0.001). Increasing histopathologic risk burden correlated with higher mortality. In the stage III cohort, ACT was administered to 66.5% of the 3044 patients and significantly improved OS (HR, 0.53; 95% CI 0.45–0.62; *p* < 0.001). Notably, one third of the patients with stage III disease did not receive ACT despite the guideline recommendations for ACT in this population.

**Conclusion:**

A patient’s MSI-H status alone should not preclude the use of ACT in colon cancer. This study demonstrated a significant survival benefit of ACT for both high-risk stage II and stage III MSI-H patients. Histopathologic risk stratification should guide treatment decisions for stage II MSI-H colon cancer, and ACT may be recommended for patients with multiple adverse pathologic features.

**Supplementary Information:**

The online version contains supplementary material available at 10.1245/s10434-025-18285-7.

Microsatellite instability (MSI) defines a distinct molecular subtype of colorectal cancer (CRC) marked by deficient DNA mismatch repair and a hypermutated genomic profile.^1^ Mismatch repair-deficient/microsatellite instability-high (dMMR/MSI-H) status, observed in approximately 10% to 20% of sporadic CRCs, has become an important biomarker influencing both prognosis and therapeutic decision-making.^2^ Generally, MSI-H tumors are associated with more favorable clinical outcomes than their microsatellite stable (MSS) counterparts.^3^

The distribution of MSI-H tumors varies by disease stage, representing a significant proportion of stage II (15–20%) and stage III (8**–**12%) CRC cases.^4^ The National Comprehensive Cancer Network^5^ (NCCN) guidelines recommend routine testing for microsatellite instability in all newly diagnosed CRCs, reflecting its growing importance in treatment planning. The clinical relevance of MSI testing has been further emphasized by the U.S. Food and Drug Administration’s approval of immune checkpoint inhibitors for metastatic MSI-H CRC.^6^ Recent clinical trials have demonstrated promising efficacy in the early-stage setting.^7–10^

Although the prognostic value of MSI status has been well-established, its predictive role for ACT benefit in stage II CRC remains controversial, largely due to limited studies incorporating histopathologic risk stratification.^4,11^ Traditionally, MSI-H tumors have been perceived as unresponsive to fluoropyrimidine-based ACT because of their distinct molecular characteristics. However, this paradigm has been challenged by randomized clinical trials demonstrating disease-free survival (DFS) and overall survival (OS) benefits of ACT in stage III CRCs regardless of MSI status. Recent discussions also have focused on optimizing the treatment duration to prevent overtreatment with potential reductions in toxicities, inconveniences, and costs associated with shorter treatment regimens.^4,7,12,13^

Current treatment guidelines differ based on disease stage and MSI status, yet a critical knowledge gap exists regarding the optimal management and potential ACT benefits when histopathologic risk stratification is incorporated, particularly for patients with stage II disease. For stage II dMMR/MSI-H colon cancer, the NCCN guidelines do not incorporate histopathologic risk features in decisions regarding adjuvant treatment.^5^ The European Society of Medical Oncology (ESMO)^14^ guidelines stratify stage II disease into low-, intermediate-, and high-risk groups. In the intermediate-risk group, MSI status plays a critical role in guiding treatment decisions. Those with MSI-H tumors are typically managed without ACT, whereas patients with MSS are more likely to receive a full 6-month course.

Whereas current guidelines predominantly recommend observation for patients with stage II MSI-H colon cancer, our study explores whether comprehensive histopathologic risk stratification could inform more personalized therapeutic decisions regarding ACT for this population. Specifically, we hypothesized that ACT may improve OS for a subset of high-risk stage II MSI-H patients who have undergone upfront surgery. To test this, we analyzed a large cohort from the National Cancer Database (NCDB) to assess the impact of ACT on survival outcomes for patients with stage II or III MSI-H colon cancer, incorporating histopathologic risk stratification.

## Methods

### Study Design

We conducted a retrospective cohort study using data from the NCDB, a hospital-based oncology registry jointly sponsored by the American College of Surgeons and the American Cancer Society. The NCDB captures approximately 70% of all newly diagnosed malignancies in the United States and includes comprehensive patient-level data from more than 1500 Commission on Cancer–accredited institutions. All data used in this analysis were de-identified and publicly available. Therefore, institutional review board approval was not required.

### Study Population

Patients with a diagnosis of stage II or III MSI-H colon adenocarcinoma (ICD-O-3 code 8140/3) between 2013 and 2019 were identified using the NCDB. The inclusion criteria specified primary tumor location from the cecum to the sigmoid colon and surgical resection. Patients were excluded if they had stage I or IV disease, non-adenocarcinoma histology, appendiceal tumors, overlapping lesions, or colon not otherwise specified (NOS), or had undergone local excision or non-specified procedures. Additionally, the study excluded patients who received neoadjuvant therapy or any form of radiation therapy. Further exclusions ruled out patients with MSS, MSI-low, unknown, or inapplicable MSI status, and patients with missing covariate data. To minimize guarantee-time bias, patients who died within 90 days postoperatively also were excluded because they would not have had the opportunity to receive ACT.^15^

The patients were categorized into two cohorts according to whether they received ACT. The stage II patients were further stratified into low- and high-risk subgroups according to the NCCN guidelines.^5^ Our study defined high-risk stage II disease by the presence of one or more of the following histopathologic features: pT4 disease, lymphovascular invasion (LVI), perineural invasion (PNI), fewer than 12 lymph nodes examined, and positive surgical margins.

### Study Outcomes and Variables

The main outcome of the study was OS in relation to ACT administration. The patients with stage II disease were stratified into high- and low-risk groups, whereas the stage III patients were analyzed as a separate cohort without additional stratification. OS was defined as the time from the date of diagnosis to death or the last known follow-up evaluation, as reported in the NCDB.

Patient demographics and clinical covariates included age at diagnosis, gender, ethnicity, Charlson-Deyo comorbidity score, and year of diagnosis. Tumor-related variables included anatomic location (right vs left colon), pathologic T stage, tumor grade, presence of LVI, PNI, number of regional lymph nodes examined, and surgical margin status.

### Statistical Analysis

Descriptive statistics were used to summarize the baseline demographic and clinical characteristics. Categorical variables are reported as frequencies and percentages and continuous variables as medians with interquartile ranges. Comparisons of baseline characteristics before and after adjustment were assessed using standardized mean differences (SMDs), with an SMD lower than 0.1 considered indicative of good covariate balance.

To reduce confounding and estimate the effect of ACT on OS, inverse probability of treatment-weighting (IPTW) based on propensity scores was applied. We selected IPTW over other propensity score methods (e.g., matching or stratification) because it allows retention of the full sample size while effectively balancing covariates between treatment groups, thereby maximizing the statistical power and generalizability of our findings. The IPTW procedure uses, weights derived from propensity scores to create a pseudo-population in which the distribution of the measured baseline covariates is independent of the treatment assignment.

Propensity scores were estimated using logistic regression models tailored to each cohort, with receipt of ACT as the dependent variable.^16^ For the low-risk stage II MSI-H cohort, the covariates included age group (≥ 65 vs < 65 years), gender, ethnicity, Charlson-Deyo comorbidity score, tumor location (right vs left colon), and year of diagnosis. For the high-risk stages II and III MSI-H cohorts, the model additionally included the pathologic T stage, LVI, PNI, number of lymph nodes examined, and surgical margin status. Kernel density plots confirmed adequate overlap in propensity scores between treatment groups after IPTW, supporting the validity of the weighted comparisons (Figs. S1 and S2).

After IPTW, OS was estimated using weighted Kaplan-Meier methods and compared between groups. Weighted Cox proportional hazards models were used to estimate hazard ratios (HRs) with 95% confidence intervals (CIs).^17^ Kaplan–Meier curves without adjustment were generated for each cohort to illustrate unadjusted outcomes (Fig. S1). For the high-risk stage II MSI-H cohort, we developed a composite risk score (range, 0–4) by summing the presence of the following key adverse features (each contributing one point): pT4 stage, LVI, PNI, positive surgical margins, and examination of fewer than 12 lymph nodes. This simple additive approach was chosen to facilitate clinical interpretation and application, with patients classified as having low (one risk factor), intermediate (two risk factors), or high (three or more risk factors) cumulative risk. Kaplan-Meier survival analysis was performed using survfit function from the survival package in R with IPTW applied to minimize confounding and evaluate OS across risk stratification. All statistical analyses were performed using the R software (R Foundation for Statistical Computing, Vienna, Austria, Version 4.2.3).

## Results

**Fig. 1:**
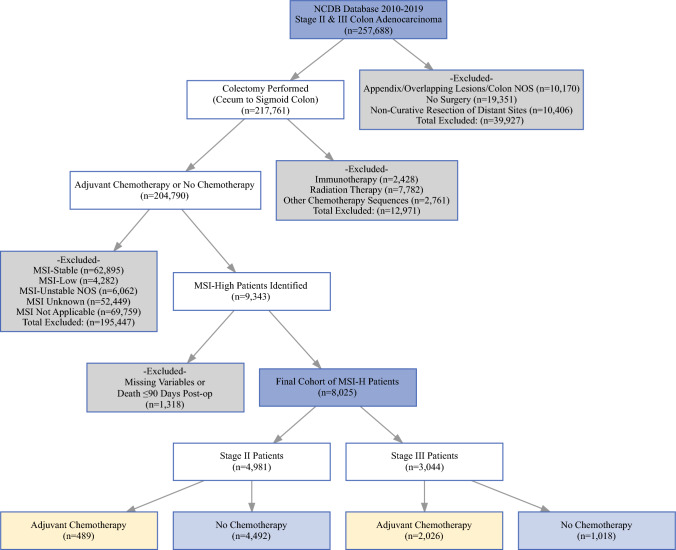
Flowchart of the patient selection.

The final cohort included 8025 patients with MSI-H colon adenocarcinoma who underwent surgery, of whom 62.1% had stage II disease and 37.9% had stage III disease (Fig. 1). Of the 8025 patients, 2515 (31.3%) received ACT, whereas 5510 (68.7%) did not. The patients who received ACT were significantly younger (median age, 66 vs 75 years; *p* < 0.001), more likely to be male (44.7% vs 36.8%; *p* < 0.001), and less likely to have right-sided tumors (79.6% vs 84.9%; *p* < 0.001). Notably, ACT was far less common among the stage II patients, with only 19.4% receiving ACT compared with 80.6% of the stage III patients. In the stage II cohort, the high-risk patients were more likely to receive ACT (57.9%) than the low-risk patients (42.1%). Additionally, high-risk pathologic features, including LVI, PNI, and positive surgical margins, were significantly more prevalent in the ACT group (all *p* < 0.001) (Table 1).

**Table 1:** Baseline demographics and pathologic characteristics of MSI-H colon cancer patients

Characteristics		No chemotherapy (*n* = 5510) *n* (%)	Adjuvant chemotherapy (*n* = 2515) *n* (%)	*p* value
Age (years)	Mean ± SD	72.6 ± 13.4	63.8 ± 14.3	< 0.01
	Median (IQR)	75 (65–83)	66 (55–75)	
Gender	Male	2025 (36.8)	1125 (44.7)	< 0.01
Ethnicity	Caucasian	4909 (89.1)	2118 (84.2)	< 0.01
	African-American	360 (6.5)	252 (10.0)	
	Other/unknown	241 (4.4)	145 (5.8)	
Charlson-Deyo score	0	3492 (63.4)	1768 (70.3)	< 0.01
	1	1083 (19.7)	463 (18.4)	
	2	488 (8.9)	150 (6.0)	
	≥ 3	447 (8.1)	134 (5.3)	
Tumor location	Left colon	830 (15.1)	512 (20.4)	< 0.01
	Right colon	4680 (84.9)	2003 (79.6)	
Stage	II	4492 (81.5)	489 (19.4)	< 0.01
	III	1018 (18.5)	2026 (80.6)	
Stage II, risk status	Low risk	3295 (73.4)	206 (42.1)	< 0.01
	High risk	1197 (26.6)	283 (57.9)	
Lymphovascular invasion	Absent	4216 (76.5)	1267 (50.4)	< 0.01
	Present	1294 (23.5)	1248 (49.6)	
Perineural invasion	Absent	5180 (94.0)	2259 (89.8)	< 0.01
	Present	330 (6.0)	256 (10.2)	
Lymph nodes retrieved	< 12	78 (1.4)	30 (1.2)	0.43
	12–16	1358 (24.6)	560 (22.3)	
	17+	4074 (73.9)	1925 (76.5)	
Surgical margin	No residual tumor	5406 (97.1)	2376 (94.5)	< 0.01
	Residual tumor	104 (1.9)	139 (5.5)	

MSI-H, microsatellite instability-high; SD, standard deviation; IQR, interquartile range

### Low-Risk Stage II Cohort

**Fig. 2:**
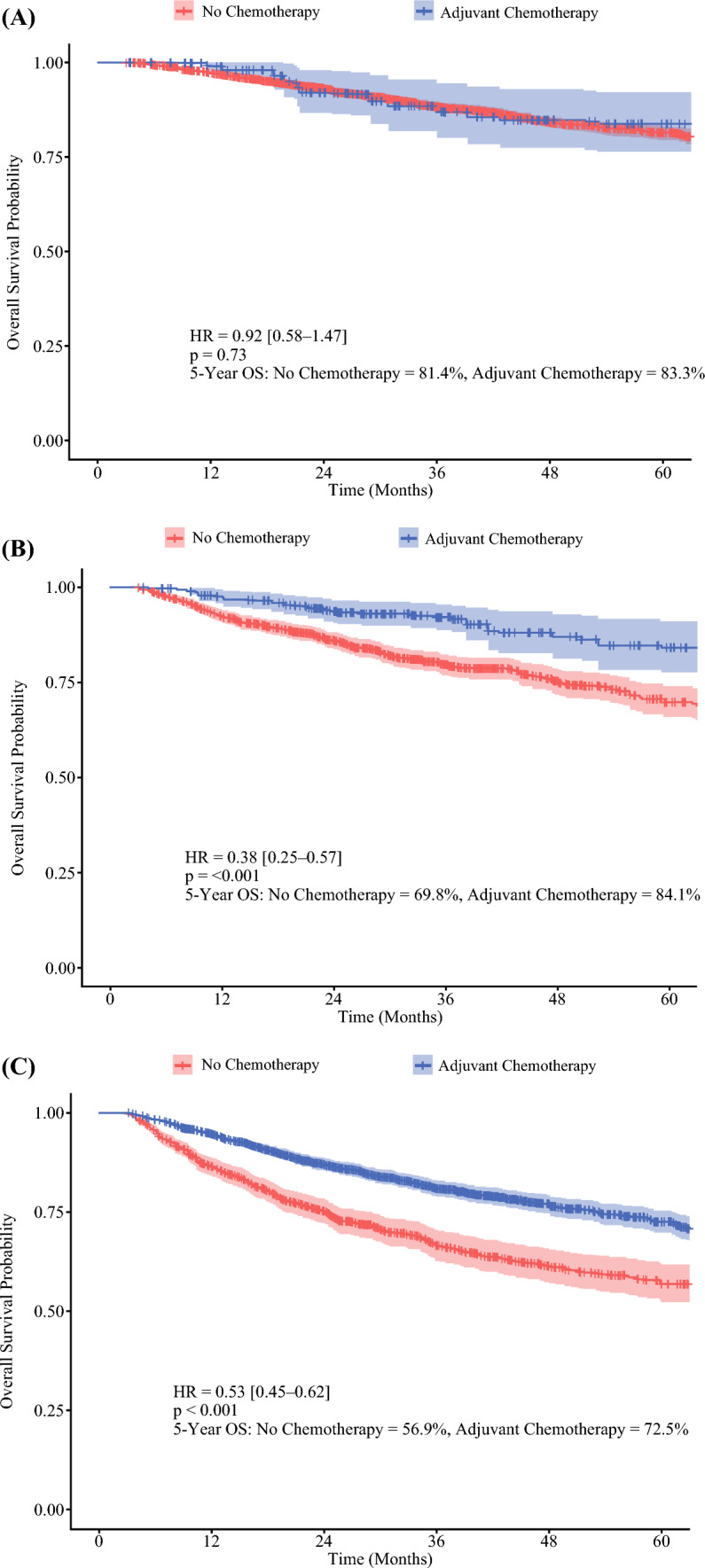
Kaplan-Meier estimates of overall survival for IPTW-adjusted cohorts. Low-risk stage II MSI-H colon cancer. High-risk stage II MSI-H colon cancer. Stage III MSI-H colon cancer. IPTW, inverse probability of treatment-weighting; MSI-H, microsatellite instability-high

Among the 3501 patients with low-risk stage II MSI-H colon cancer, only 206 (5.9%) received ACT, whereas the vast majority (*n* = 3295, 94.1%) underwent observation alone. Before IPTW adjustment, the ACT recipients were significantly younger and more likely to be male, with a higher proportion having left-sided tumors (Table 2). After IPTW adjustment to balance baseline characteristics, the 5-year OS showed no significant difference between the ACT (83.3%) and no chemotherapy (81.4%) groups (HR, 0.92; 95% CI 0.58–1.47; *p* = 0.73; Fig. 2A).

**Table 2:** Baseline characteristics of patients with low-risk stage II MSI-H colon cancer before and after IPTW adjustment

Characteristics		Low-risk patient cohort						
		Unadjusted			IPTW-adjusted			
		No chemotherapy (*n* = 3295) *n* (%)	Adjuvant chemotherapy (*n* = 206) *n* (%)	*p* value	No chemotherapy	Adjuvant chemotherapy	*p* value	SMD
Age (years)	< 65	962 (29.2)	127 (61.7)	< 0.001	(24.9)	(23.8)	0.71	0.03
	≥ 65	2333 (70.8)	79 (38.3)		(75.1)	(76.2)		
Gender	Male	1298 (39)	111 (54)	< 0.001	(37.5)	(35.8)	0.67	0.04
Ethnicity	Caucasian	2926 (88.8)	176 (85.4)	0.49	(88.8)	(89.5)	0.93	0.06
	African-American	224 (6.8)	19 (9.2)		(4.7)	(5.5)		
	Other/unknown	145 (3.9)	149 (71.8)		(4.4)	(5)		
Charlson-Deyo score	0	2124 (64.5)	148 (71.8)	0.19	(65)	(67.6)	0.91	0.07
	1	654 (19.8)	33 (16.0)		(19)	(16.4)		
	2	270 (8.2)	12 (5.8)		(8.2)	(8.6)		
	≥ 3	247 (7.5)	13 (6.3)		(7.8)	(7.4)		
Tumor location	Left colon	520 (15.8)	57 (27.7)	< 0.01	(14.5)	(12.6)	0.47	0.06
	Right colon	2775 (84.2)	149 (72.3)		(85.5)	(87.4)		

MSI-H, microsatellite instability-high; IPTW, inverse probability of treatment-weighting; SMD, standardized mean difference (between groups after weighting, used to assess covariate balance)

### High-Risk Stage II Cohort

Of the 1480 high-risk stage II patients, 1197 (80.9%) did not receive ACT despite their high-risk profile. Only 283 (19.1%) received ACT. Before IPTW adjustment, the ACT recipients were significantly younger, male, and more likely to present with pT4 tumors and adverse histopathologic features (Table 3). After IPTW adjustment to minimize selection bias, ACT was associated with a significant improvement in the 5-year OS (84.1% vs 69.8%; HR, 0.38; 95% CI 0.25–0.57; *p* < 0.001; Fig. 2B), corresponding to a 62% reduction in mortality risk.

**Table 3:** Baseline characteristics of patients with high-risk stage II MSI-H colon cancer before and after IPTW adjustment

Characteristics		High-risk patient cohort						
		Unadjusted			IPTW-adjusted			
		No chemotherapy (*n* = 1197) *n* (%)	Adjuvant chemotherapy (*n* = 283) *n* (%)	*p* value	No chemotherapy	Adjuvant chemotherapy	*p* value	SMD
Age (years)	< 65	225 (18.8)	143 (50.5)	< 0.001	(14.9)	(13.7)	0.69	0.03
	≥ 65	972 (81.2)	140 (49.5)		(75.1)	(76.3)		
Gender	Male	408 (34.1)	146 (51.6)	< 0.001	(37.5)	(36.2)	0.74	0.03
Ethnicity	Caucasian	1073 (89.6)	241 (85.2)	0.17	(88.8)	(90)	0.87	0.06
	African-American	71 (5.9)	24 (8.5)		(6.5)	(5.2)		
	Other/unknown	53 (4.5)	18 (6.4)		(4.7)	(4.8)		
Charlson-Deyo score	0	9 (0.8)	202 (71.4)	< 0.001	(65)	(67.5)	0.9	0.07
	1	756 (63.2)	59 (20.8)		(19)	(16.2)		
	2	226 (18.9)	12 (4.2)		(8.2)	(8.6)		
	≥ 3	109 (9.1)	10 (3.5)		(7.8)	(7.6)		
Tumor location	Left colon	165 (13.8)	52 (18.4)	0.06	(92.2)	(92.4)	0.54	0.05
	Right colon	1032 (86.2)	231 (81.6)		(7.8)	(7.6)		
pT stage	pT3	759 (63.4)	98 (34.6)	< 0.001	(57.7)	(57.4)	0.96	0.04
	pT4	438 (36.6)	185 (65.4)		(42.3)	(42.6)		
Lymphovascular invasion	Absent	477 (39.8)	158 (55.8)	< 0.001	(42.7)	(41.2)	0.73	0.03
	Present	720 (60.2)	125 (44.2)		(57.3)	(58.8)		
Perineural invasion	Absent	1013 (84.6)	258 (91.2)	0.006	(85.9)	(85.3)	0.86	0.02
	Present	184 (15.4)	25 (8.8)		(14.1)	(14.7)		
Lymph nodes retrieved	< 12	57 (4.8)	8 (2.8)	0.16	(4.3)	(2.2)	0.32	0.12
	12–16	261 (21.8)	73 (25.8)		(22.5)	(23.2)		
	17+	879 (73.4)	202 (71.4)		(73.2)	(74.6)		
Surgical margin	No residual tumor	1132 (94.6)	249 (88.0)	< 0.001	(93.5)	(93.9)	0.8	0.02
	Residual tumor	65 (5.4)	34 (12.0)		(6.5)	(6.1)		

MSI-H, microsatellite instability-high; IPTW, inverse probability of treatment-weighting; SMD, standardized mean difference (between groups after weighting, used to assess covariate balance)

**Fig. 3:**
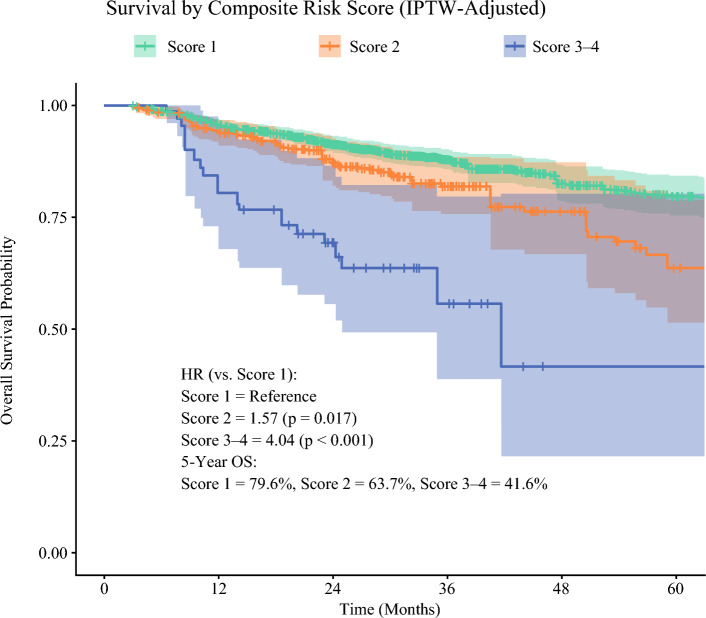
IPTW-adjusted Kaplan-Meier plot for overall survival in high-risk stage II MSI-H colon cancer. Patients were categorized by a composite risk score based on high-risk features: lymphovascular invasion (LVI), perineural invasion (PNI), T4 stage, positive surgical margins, or fewer than 12 lymph nodes examined. Score 1 indicates one risk factor, whereas score 2 indicates two risk factors, and scores 3 and 4 indicate three and four risk factors. HR, hazard ratio; OS, overall survival; IPTW, inverse probability of treatment-weighting; MSI-H, microsatellite instability-high

Further analysis of the cumulative pathologic risk demonstrated a significant stepwise decrease in OS with increasing risk factor burden. The patients with two risk factors exhibited a mortality risk nearly double that of the patients with a single risk factor. Notably, those with three or more high-risk features had a fourfold increase in mortality risk, highlighting a strong association between cumulative histopathologic risk burden and inferior survival outcomes (Fig. 3).

To further explore the impact of chemotherapy intensity, we stratified ACT recipients by regimen in the unadjusted cohort. Among the 283 patients who received ACT, 92 (32.5%) received single-agent therapy, 176 (62.2%) received multi-agent therapy, and 15 (5.3%) had undocumented regimen details. Compared with the patients who received no chemotherapy, those with single-agent therapy showed an a non-significant trend toward improved survival (HR, 0.6; 95% CI 0.36–1.01; *p* = 0.056), whereas multi-agent therapy was associated with a significantly reduced mortality risk (HR, 0.39; 95% CI 0.25–0.62; *p* < 0.001) (Fig. S3).

### Stage III Cohort

The stage III cohort comprised 3044 patients, with 2026 (66.5%) receiving ACT and 1018 (33.5%) managed with observation. The recipients of ACT were significantly younger and more likely to have left-sided tumors (Table 4). After IPTW adjustment, ACT was associated with a substantially improved 5-year OS (72.5% vs 56.9%; HR, 0.53; 95% CI 0.45–0.62; *p* < 0.001; Fig. 2C).

**Table 4:** Baseline characteristics of patients with stage III MSI-H colon cancer before and after IPTW adjustment

Characteristics		Unadjusted			IPTW-adjusted			
		No chemotherapy (*n* = 1018) *n* (%)	Adjuvant chemotherapy (*n* = 2026) *n* (%)	*p* value	No chemotherapy	Adjuvant chemotherapy	*p* value	SMD
Age (years)	< 65	132 (13.0)	896 (44.2)	< 0.001	(34.5)	(33.8)	0.77	0.02
	≥ 65	886 (87.0)	1130 (55.8)		(65.5)	(66.2)		
Gender	Male	319 (31.3)	868 (42.8)	< 0.001	(42.4)	(39.3)	0.17	0.06
Ethnicity	Caucasian	910 (89.4)	1701 (84.0)	0.001	(85.6)	(85.8)	0.89	0.04
	African-American	65 (6.4)	209 (10.3)		(9.9)	(9.1)		
	Other/unknown	43 (4.2)	116 (5.7)		(4.5)	(5.2)		
Charlson-Deyo score	0	612 (60.1)	1418 (70.0)	< 0.001	(68.1)	(66.8)	0.89	0.03
	1	203 (19.9)	371 (18.3)		(17.7)	(18.7)		
	2	109 (10.7)	126 (6.2)		(7.5)	(7.7)		
	≥ 3	94 (9.2)	111 (5.5)		(6.7)	(6.7)		
Tumor location	Left colon	145 (14.2)	403 (19.9)	< 0.001	(18.0)	(19.0)	0.99	< 0.001
	Right colon	873 (85.8)	1623 (80.1)		(82.0)	(82.0)		
pT stage	non-pT4	755 (74.2)	1537 (75.9)	0.33	(86.6)	(85.5)	0.55	0.03
	pT4	263 (25.8)	489 (24.1)		(23.4)	(24.5)		
Lymphovascular invasion	Absent	444 (43.6)	903 (44.6)	0.64	(45.3)	(44.4)	0.67	0.02
	Present	574 (56.4)	1123 (55.4)		(54.7)	(55.6)		
Perineural invasion	Absent	872 (85.7)	1795 (88.6)	0.02	(87.0)	(87.5)	0.71	0.02
	Present	146 (14.3)	231 (11.4)		(13.0)	(12.5)		
Lymph nodes retrieved	< 12	21 (2.1)	22 (1.1)	< 0.001	(1.2)	(1.3)	0.95	0.01
	12–16	282 (27.7)	445 (22.0)		(24.1)	(23.9)		
	17+	715 (70.2)	1559 (76.9)		(74.7)	(74.7)		
Surgical margin	No residual tumor	979 (96.2)	1921 (94.8)	0.12	(95.9)	(95.3)	0.52	0.03
	Residual tumor	39 (3.8)	105 (5.2)		(4.1)	(4.7)		

MSI-H, microsatellite instability-high; IPTW, inverse probability of treatment-weighting; SMD, standardized mean difference (between groups after weighting, used to assess covariate balance)

## Discussion

In this large U.S. nationwide analysis of 8025 patients with stage II or III MSI-H colon cancer, ACT was associated with a substantial OS benefit for stage III and high-risk stage II patients, but not for low-risk stage II patients. After adjustment for IPTW, our findings suggested that risk stratification using histopathologic features should guide ACT decisions in MSI-H stage II colon cancer rather than a uniform approach or omission of ACT based solely on the MSI-H subtype. This is consistent with a recent Korean study of 158 MSI-H stage II patients, which demonstrated significantly improved OS and disease-free survival with ACT for patients with high-risk features, supporting the recommendation for this population to receive ACT regardless of MSI status.^18^

Additionally, it is important for clinicians to consider composite risk score when deciding on ACT for patients with MSI-H stage II disease. Our findings showed that patients with two risk factors exhibited a mortality risk nearly double that for those with a single risk factor, whereas those with three or more high-risk features faced a fourfold increase in mortality risk, highlighting a strong association between cumulative histopathologic risk burden and inferior survival outcomes.

The 2024 NCCN Colon Cancer Guidelines^5^ recommend observation rather than ACT for patients with stage IIA or IIB MSI-H/dMMR colon cancer. For stage IIC MSI-H/dMMR disease, either observation or adjuvant chemotherapy may be considered, using the same treatment protocols recommended for patients with low-risk stage III disease. In contrast to its approach for pMMR/MSS stage II tumors, in which specific high-risk features (e.g., inadequate lymph node sampling, LVI, PNI, bowel obstruction, poor differentiation, or positive margins) guide ACT recommendations, the NCCN does not currently stratify patients with stage II MSI-H/dMMR patients based on clinicopathologic risk factors. In addition, the ESMO guidelines^14^ stratify stage II colon cancer into low-, intermediate-, and high-risk categories. For intermediate-risk patients (those with one factor such as LVI, PNI, tumor obstruction, elevated preoperative CEA, vascular invasion, or poor histologic grade), observation is recommended if the tumor is MSI-H. However, for patients classified as high risk, including those with pT4 tumors, inadequate lymph node evaluation (< 12 nodes), or multiple intermediate-risk features, ACT is recommended regardless of the MSI status.

These recommendations are based largely on earlier studies that compared treatment outcomes based on MSI status in non-metastatic CRCs. The prognostic value of MSI status has been extensively studied, with many studies and meta-analyses demonstrating that MSI-H status is associated with better prognosis than MSS status in stage II and III CRCs.^19–22^ The traditional clinical approach has favored ACT for MSS tumors while questioning its benefit in MSI-H cases, based on observations that MSI-H tumors show reduced recognition of DNA adducts by the DNA MMR system, potentially limiting chemotherapy effectiveness.^22^ However, our findings challenge this perspective by demonstrating significant OS benefit with ACT for high-risk MSI-H stage II patients, suggesting that histopathologic risk stratification rather than MSI status alone should guide treatment decisions.

A distinct stage distribution is demonstrated by MSI-H tumors. being more common in stage II disease (15–20%) than in stage III disease (8-12%) and significantly less common in stage IV tumors (3.5%). This distribution suggests decreased metastatic potential in MSI-H CRCs.^2,4,5,23^ Our study cohort aligns with these patterns, with 62% of MSI-H colon adenocarcinoma patients presenting with stage II disease and 38% with stage III disease after surgical resection.

Despite evidence supporting the benefit of ACT for select populations, our analysis showed patterns of potentially suboptimal ACT utilization among the high-risk stages II and III MSI-H patients. Notably, only 66.6% of the stage III MSI-H patients received ACT despite guideline recommendations for ACT in stage III disease regardless of MSI status. Several clinical factors may influence treatment variation, including provider uncertainty about the benefit-risk balance, comorbidities, patient preferences, and socioeconomic barriers. In addition, positive surgical margins, although relatively uncommon in colon cancer resections, as shown in our data, represent a particularly concerning high-risk feature. These findings highlight the importance of achieving R0 resection when technically feasible and suggest that margin status should be considered in the risk stratification framework for ACT decision-making for MSI-H patients. However, due to limitations inherent to retrospective database analysis, we were unable to identify the specific reasons driving treatment decisions in individual cases.

Although our findings demonstrated favorable outcomes with ACT for high-risk MSI-H subgroups, the potential for overtreatment and associated toxicity requires careful consideration. Fluoropyrimidine- and oxaliplatin-based regimens have significant toxic effects and long-term quality-of-life impairments. Some studies suggest decreased benefit or even detrimental effects of fluoropyrimidine monotherapy for stage II disease but not for stage III disease. However, other studies have demonstrated that although MSI status is prognostic, it does not predict benefit or harm from ACT.^22,24–26^ A recently published updated meta-analysis confirmed no detrimental effect for stage III colon cancer patients.^19^ Therefore, for low-risk stage II patients with MSI-H tumors who already demonstrate favorable baseline survival, the absolute benefit of ACT is likely minimal and may not outweigh these harmful effects. Regarding ACT regimen selection for high-risk stage II patients, a recently published Korean study found no significant difference in outcomes between those receiving fluorouracil (5-FU)-based ACT and those receiving oxaliplatin-based ACT. These findings suggest that when ACT is indicated for stage II MSI-H patients, 5-FU-based regimens may provide comparable efficacy with potentially lower toxicity than oxaliplatin-based therapy.^18^

This study has some limitations inherent to retrospective database analyses. The NCDB lacks detailed information on chemotherapy agents, recurrence, and disease-free survival, which limited the scope of our survival interpretation. Furthermore, heterogeneity in institutional practices regarding MSI testing methods and treatment protocols may have influenced our results. Additionally, patient preferences, comorbidities, and provider-specific approaches may have contributed to these observations, but they could not be fully assessed within our dataset.

Nevertheless, our study had several strengths, including its large national database that provides a robust sample size and helps to reduce sampling bias. This improves the generalizability of the findings, in contrast to single-center studies, and allowed us to apply rigorous statistical adjustment using IPTW to reduce selection bias. Stratification by histopathologic risk enables a more granular understanding of the benefits of ACT for MSI-H patients.

Although this study provides important insights into the impact of adjuvant chemotherapy for patients with stage II or III MSI-H colon cancer who underwent upfront surgery, it does not account for the rapidly evolving role of immunotherapy. Although immunotherapy has become the standard of care for metastatic MSI-H disease and currently is being incorporated into adjuvant treatment guidelines for locally advanced colon cancer, chemotherapy remains a commonly used option in real-world settings. According to the NCCN version 4.2025 guidelines, adjuvant immunotherapy regimens such as FOLFOX combined with atezolizumab are currently listed as category 2B recommendations (based on lower-level evidence without uniform consensus) for patients with stage IIC or III MSI-H colon cancer.^27^ Moreover, neoadjuvant strategies are gaining momentum, with recent studies of neoadjuvant immunotherapy reporting unprecedented response rates compared with the 7% pathologic response seen with neoadjuvant chemotherapy for MSI-H tumors in the FOxTROT trial.^9,10^ However, real-world adoption of these newer approaches remains limited due to factors such as cost, infrastructure availability, and patient selection criteria.

Our analysis excluded patients who received immunotherapy during the study period, which represented a significant limitation. However, we did not include this subgroup due to several methodologic challenges. The immunotherapy patients were highly heterogeneous, with limited granularity on treatment dosing, duration, and timing. Furthermore, this subgroup had incomplete data on key histopathologic variables used in our risk stratification analysis. Including these patients would have substantially limited statistical power and introduced the risk of unreliable survival estimates.

Until immunotherapy becomes widely accessible and established as the standard of care across institutions, our findings remain highly relevant for patients undergoing upfront surgery. Should immunotherapy gain widespread adoption supported by high-level evidence and expert consensus, the role of risk stratification in this population may need to be reevaluated within future immunotherapy-based treatment paradigms.

## Conclusion

In colon cancer, MSI-H status alone should not serve as the sole determinant of ACT decisions. Although low-risk stage II MSI-H patients appear to derive minimal survival benefits from ACT, both high-risk stages II and III patients with MSI-H tumors experience significant improvements in OS with ACT. Importantly, the cumulative burden of histopathologic risk factors is strongly associated with increased mortality for high-risk patients with stage II MSI-H. Future prospective studies to validate these findings are warranted, with a focus on identifying which stage II MSI-H patients benefit most from treatment and weighing the survival benefits of chemotherapy against its potential toxicity.

## Supplementary Information

Below is the link to the electronic supplementary material.

Supplementary file1 (DOCX 1053 kb)

Supplementary file1 (DOCX 861 kb)

Supplementary file1 (DOCX 137 kb)

## References

[1] Poynter JN, Siegmund KD, Weisenberger DJ, et al. Molecular characterization of MSI-H colorectal cancer by MLHI promoter methylation, immunohistochemistry, and mismatch repair germline mutation screening. *Cancer Epidemiol Biomarkers Prev*. 2008;17:3208–15. 10.1158/1055-9965.EPI-08-0512. 18990764 10.1158/1055-9965.EPI-08-0512 PMC2628332

[2] Koopman M, Kortman GAM, Mekenkamp L, et al. Deficient mismatch repair system in patients with sporadic advanced colorectal cancer. *Br J Cancer*. 2009;100:266–73. 10.1038/sj.bjc.6604867. 19165197 10.1038/sj.bjc.6604867 PMC2634718

[3] Kang S, Na Y, Joung SY, Lee SI, Oh SC, Min BW. The significance of microsatellite instability in colorectal cancer after controlling for clinicopathological factors. *Med Baltim*. 2018;97:e0019. 10.1097/MD.0000000000010019. 10.1097/MD.0000000000010019 PMC5851768 29489646

[4] Taieb J, André T, Auclin E. Refining adjuvant therapy for non-metastatic colon cancer, new standards and perspectives. *Cancer Treat Rev*. 2019;75:1–11. 10.1016/j.ctrv.2019.02.002. 30849607 10.1016/j.ctrv.2019.02.002

[5] Benson AB, Venook AP, Adam M, et al. Colon cancer, version 3.2024, NCCN Clinical Practice Guidelines in Oncology. *J Natl Compr Canc Netw*. 2024;22:e240029. 10.6004/jnccn.2024.0029. 38862008 10.6004/jnccn.2024.0029

[6] Research C for DE and FDA approves nivolumab with ipilimumab for unresectable or metastatic MSI-H or dMMR colorectal cancer. *FDA*. Published online 4 August 2025. Retrieved 19 April 2025 at https://www.fda.gov/drugs/resources-information-approved-drugs/fda-approves-nivolumab-ipilimumab-unresectable-or-metastatic-msi-h-or-dmmr-colorectal-cancer.

[7] Sun BL. Current microsatellite instability testing in management of colorectal cancer. *Clin Colorect Cancer*. 2021;20:e12–20. 10.1016/j.clcc.2020.08.001. 10.1016/j.clcc.2020.08.001 32888812

[8] Chalabi M, Fanchi LF, Van Den Berg JG, et al. Neoadjuvant ipilimumab plus nivolumab in early-stage colon cancer. *Ann Oncol*. 2018;29:731. 10.1093/annonc/mdy424.047.29236943

[9] Verschoor YL, van den Berg J, Beets G, et al. Neoadjuvant nivolumab, ipilimumab, and celecoxib in MMR-proficient and MMR-deficient colon cancers: Final clinical analysis of the NICHE study. *J Clin Oncol*. 2022;40(16_suppl):3511. 10.1200/JCO.2022.40.16_suppl.3511.

[10] Morton D, Seymour M, Magill L, et al. Preoperative chemotherapy for operable colon cancer: mature results of an international randomized controlled trial. *J Clin Oncol*. 2023;41:1541–52. 10.1200/JCO.22.00046. 36657089 10.1200/JCO.22.00046 PMC10022855

[11] Evrard C, Tachon G, Randrian V, Karayan-Tapon L, Tougeron D. Microsatellite instability: diagnosis, heterogeneity, discordance, and clinical impact in colorectal cancer. *Cancers Basel*. 2019;11:1567. 10.3390/cancers11101567. 31618962 10.3390/cancers11101567 PMC6826728

[12] Sinicrope FA, Foster NR, Thibodeau SN, et al. DNA mismatch repair status and colon cancer recurrence and survival in clinical trials of 5-fluorouracil-based adjuvant therapy. *J Natl Cancer Inst*. 2011;103:863–75. 10.1093/jnci/djr153. 21597022 10.1093/jnci/djr153 PMC3110173

[13] André T, Meyerhardt J, Iveson T, et al. Effect of duration of adjuvant chemotherapy for patients with stage III colon cancer (IDEA collaboration): final results from a prospective, pooled analysis of six randomised, phase 3 trials. *Lancet Oncol*. 2020;21:1620–9. 10.1016/S1470-2045(20)30527-1. 33271092 10.1016/S1470-2045(20)30527-1 PMC7786835

[14] Argilés G, Tabernero J, Labianca R, et al. Localised colon cancer: ESMO Clinical Practice Guidelines for diagnosis, treatment and follow-up†. *Ann Oncol*. 2020;31:1291–305. 10.1016/j.annonc.2020.06.022. 32702383 10.1016/j.annonc.2020.06.022

[15] Giobbie-Hurder A, Gelber RD, Regan MM. Challenges of guarantee-time bias. *J Clin Oncol*. 2013;31:2963–9. 10.1200/JCO.2013.49.5283. 23835712 10.1200/JCO.2013.49.5283 PMC3732313

[16] Austin PC. An introduction to propensity score methods for reducing the effects of confounding in observational studies. *Multivar Behav Res*. 2011;46:399–424. 10.1080/00273171.2011.568786. 10.1080/00273171.2011.568786 PMC3144483 21818162

[17] Austin PC. The use of propensity score methods with survival or time-to-event outcomes: reporting measures of effect similar to those used in randomized experiments. *Stat Med*. 2014;33:1242–58. 10.1002/sim.5984. 24122911 10.1002/sim.5984 PMC4285179

[18] Bae SU, Lee JL, Yang CS, et al. Survival benefit of adjuvant chemotherapy in high-risk patients with colon cancer regardless of microsatellite instability. *Eur J Surg Oncol*. 2025;51:109674. 10.1016/j.ejso.2025.109674. 40043595 10.1016/j.ejso.2025.109674

[19] Cohen R, Taieb J, Fiskum J, et al. Microsatellite instability in patients with stage III colon cancer receiving fluoropyrimidine with or without oxaliplatin: an ACCENT pooled analysis of 12 adjuvant trials. *JCO*. 2021;39:642–51. 10.1200/JCO.20.01600. 10.1200/JCO.20.01600 PMC8189604 33356421

[20] Ribic CM, Sargent DJ, Moore MJ, et al. Tumor microsatellite-instability status as a predictor of benefit from fluorouracil-based adjuvant chemotherapy for colon cancer. *N Engl J Med*. 2003;349:247–57. 10.1056/NEJMoa022289. 12867608 10.1056/NEJMoa022289 PMC3584639

[21] André T, Boni C, Navarro M, et al. Improved overall survival with oxaliplatin, fluorouracil, and leucovorin as adjuvant treatment in stage II or III colon cancer in the MOSAIC trial. *J Clin Oncol*. 2009;27:3109–16. 10.1200/JCO.2008.20.6771. 19451431 10.1200/JCO.2008.20.6771

[22] Sargent DJ, Marsoni S, Monges G, et al. Defective mismatch repair as a predictive marker for lack of efficacy of fluorouracil-based adjuvant therapy in colon cancer. *J Clin Oncol*. 2010;28:3219–26. 10.1200/JCO.2009.27.1825. 20498393 10.1200/JCO.2009.27.1825 PMC2903323

[23] Roth AD, Tejpar S, Delorenzi M, et al. Prognostic role of KRAS and BRAF in stage II and III resected colon cancer: results of the translational study on the PETACC-3, EORTC 40993, SAKK 60–00 trial. *J Clin Oncol*. 2010;28:466–74. 10.1200/JCO.2009.23.3452. 20008640 10.1200/JCO.2009.23.3452

[24] Kim JE, Hong YS, Kim HJ, et al. Defective mismatch repair status was not associated with DFS and OS in stage II colon cancer treated with adjuvant chemotherapy. *Ann Surg Oncol*. 2015;22(Suppl 3):S630–7. 10.1245/s10434-015-4807-6. 26271397 10.1245/s10434-015-4807-6

[25] Hutchins G, Southward K, Handley K, et al. Value of mismatch repair, KRAS, and BRAF mutations in predicting recurrence and benefits from chemotherapy in colorectal cancer. *J Clin Oncol*. 2011;29:1261–70. 10.1200/JCO.2010.30.1366. 21383284 10.1200/JCO.2010.30.1366

[26] Bertagnolli MM, Redston M, Compton CC, et al. Microsatellite instability and loss of heterozygosity at chromosomal location 18q: prospective evaluation of biomarkers for stages II and III colon cancer: a study of CALGB 9581 and 89803. *J Clin Oncol*. 2011;29:3153–62. 10.1200/JCO.2010.33.0092. 21747089 10.1200/JCO.2010.33.0092 PMC3157981

[27] National Comprehensive Cancer Network. (2025). Colon Cancer (Version 4.2025). NCCN Clinical Practice Guidelines in Oncology. 10.6004/jnccn.2025.0017 40203873

