# Supplementary material for: Adjuvant Chemotherapy and Survival in MSI-High Stages II and III Colon Cancer: Impact of Histopathologic Risk Stratification
Source: Ann Surg Oncol. 2025 Oct 7;32(13):9510–20. doi: 10.1245/s10434-025-18285-7 (PMC12589266; doi:10.1245/s10434-025-18285-7)
Supplement: Supplementary file 1 — Supplementary file1 (DOCX 1053 kb) [file 10434_2025_18285_MOESM1_ESM.docx]

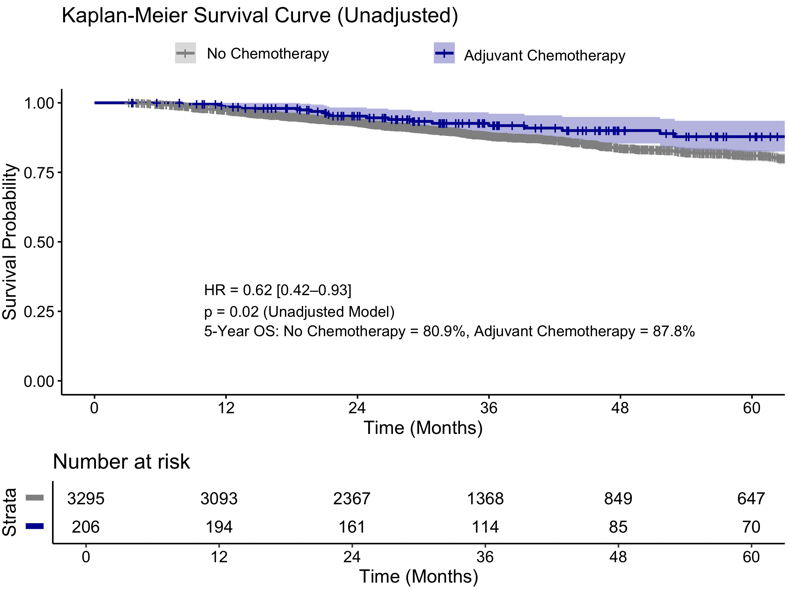

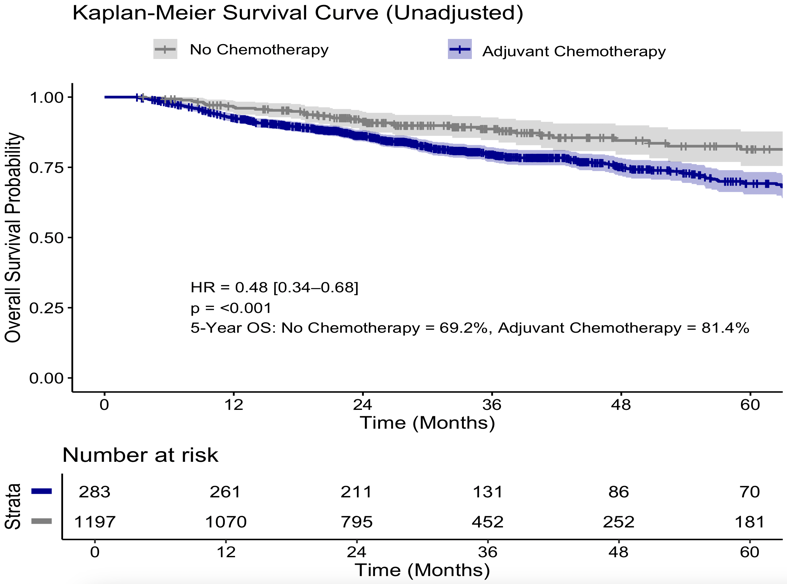

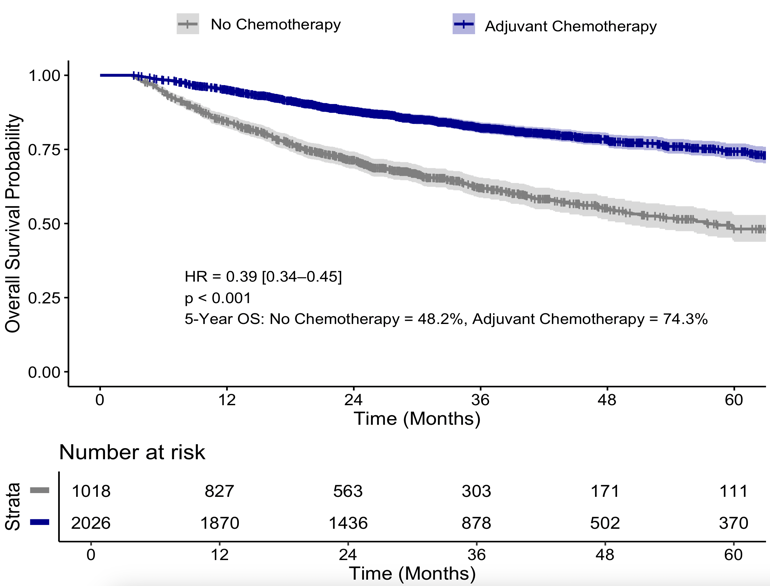


(A)

(B)

(C)

| **Supplemental Figure 1.** Kaplan-Meier Estimates of Overall Survival for Unadjusted Cohorts  A) Low-Risk Stage II MSI-H Colon Cancer  B) High-Risk Stage II MSI-H Colon Cancer  C) Stage III MSI-H Colon Cancer |
| --- |
