# Supplementary material for: Adjuvant Chemotherapy and Survival in MSI-High Stages II and III Colon Cancer: Impact of Histopathologic Risk Stratification
Source: Ann Surg Oncol. 2025 Oct 7;32(13):9510–20. doi: 10.1245/s10434-025-18285-7 (PMC12589266; doi:10.1245/s10434-025-18285-7)
Supplement: Supplementary file 2 — Supplementary file1 (DOCX 861 kb) [file 10434_2025_18285_MOESM2_ESM.docx]

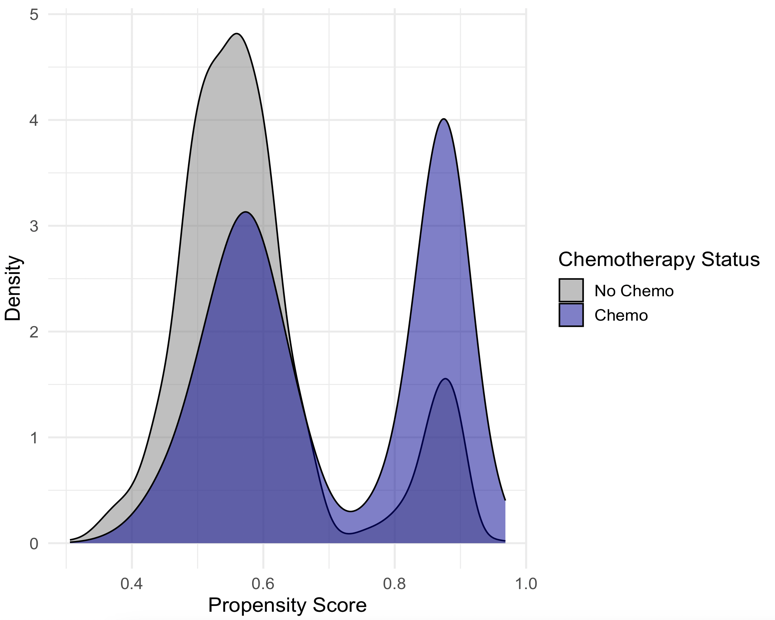

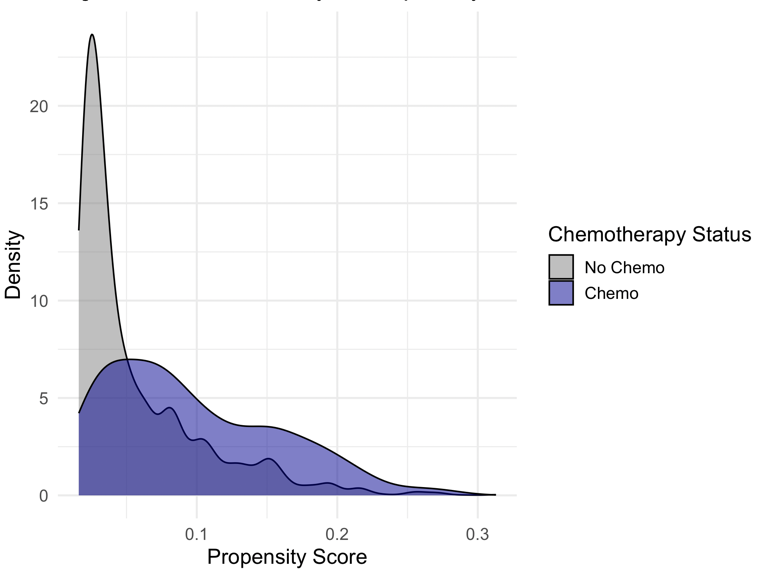


(B)

(C)


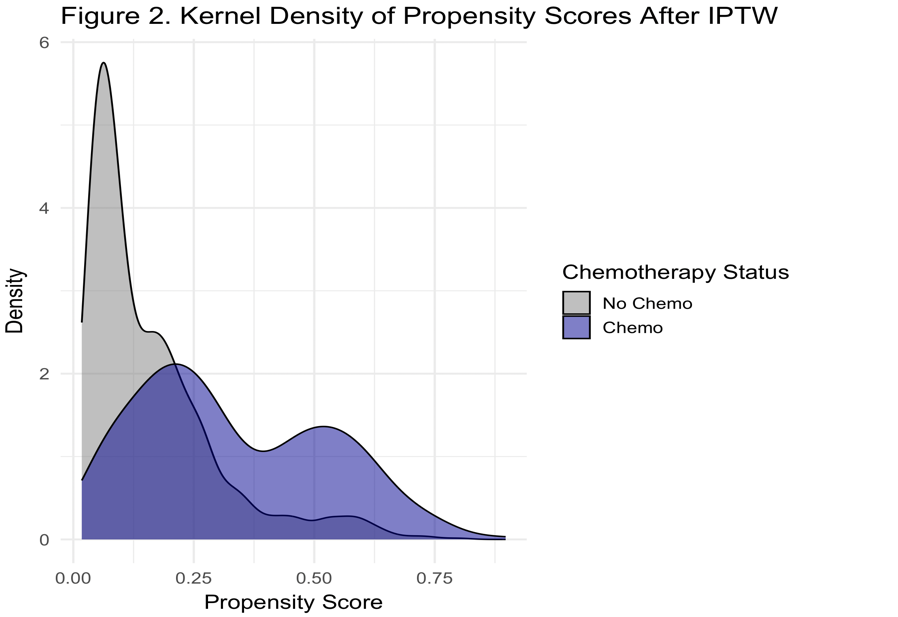


(A)

| **Supplemental Figure 2.** Kernel density plot showing the distribution of propensity scores in the adjuvant chemotherapy group vs no chemotherapy group  A) Low-Risk Stage II MSI-H Colon Cancer  B) High-Risk Stage II MSI-H Colon Cancer  C) Stage III MSI-H Colon Cancer |
| --- |
