# Supplementary material for: Adjuvant Chemotherapy and Survival in MSI-High Stages II and III Colon Cancer: Impact of Histopathologic Risk Stratification
Source: Ann Surg Oncol. 2025 Oct 7;32(13):9510–20. doi: 10.1245/s10434-025-18285-7 (PMC12589266; doi:10.1245/s10434-025-18285-7)
Supplement: Supplementary file 3 — Supplementary file1 (DOCX 137 kb) [file 10434_2025_18285_MOESM3_ESM.docx]

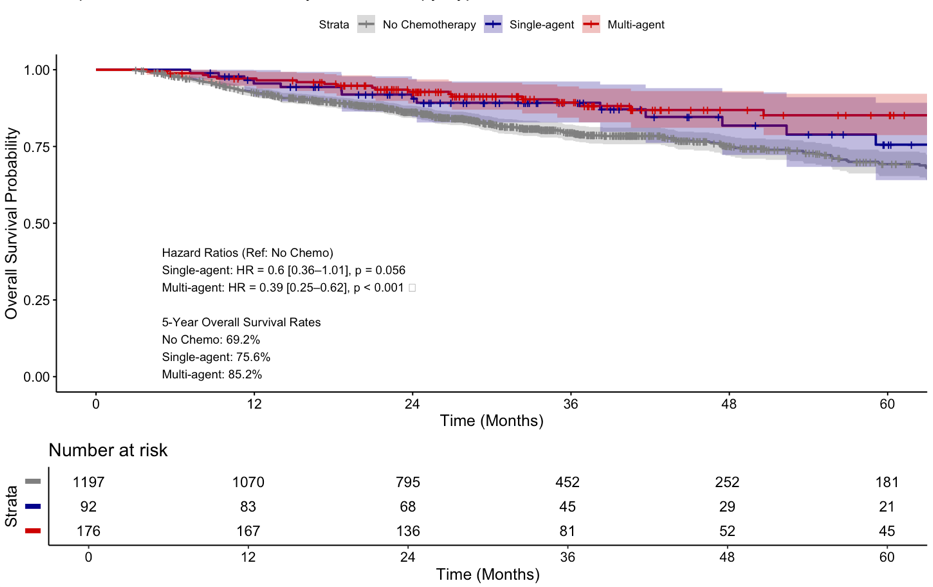


| **Supplemental Figure 3** Kaplan-Meier Estimates of Overall Survival in High-Risk Stage II MSI-High Colon Cancer Stratified by Chemotherapy Type  *Fifteen patients were excluded from this analysis due to undocumented chemotherapy type and agent number.* |
| --- |
